# Novel Crown Ether Amino Acids as Fluorescent Reporters for Metal Ions

**DOI:** 10.3390/molecules28083326

**Published:** 2023-04-09

**Authors:** Patrícia M. R. Batista, Cátia D. F. Martins, M. Manuela M. Raposo, Susana P. G. Costa

**Affiliations:** Centre of Chemistry, Campus de Gualtar, University of Minho, 4710-057 Braga, Portugal

**Keywords:** crown ether, benzoxazole, unnatural amino acids, recognition, palladium, fluorimetric chemosensors

## Abstract

Unnatural amino acids with enhanced properties, such as increased complexing ability and luminescence, are considered to be highly attractive building blocks for bioinspired frameworks, such as probes for biomolecule dynamics, sensitive fluorescent chemosensors, and peptides for molecular imaging, among others. Therefore, a novel series of highly emissive heterocyclic alanines bearing a benzo[*d*]oxazolyl unit functionalized with different heterocyclic π-spacers and (aza)crown ether moieties was synthesized. The new compounds were completely characterized using the usual spectroscopic techniques and evaluated as fluorimetric chemosensors in acetonitrile and aqueous mixtures in the presence of various alkaline, alkaline-earth, and transition metal ions. The different crown ether binding moieties as well as the electronic nature of the π-bridge allowed for fine tuning of the sensory properties of these unnatural amino acids towards Pd^2+^ and Fe^3+^, as seen by spectrofluorimetric titrations.

## 1. Introduction

Synthetic unnatural amino acids can be a source of structural diversity and functional versatility and are often used as building blocks and molecular scaffolds in the construction of peptide and proteins with tailored properties [1,2,3,4,5,6,7,8,9,10,11,12,13,14,15,16,17]. Recently, systems incorporating unnatural amino acids have been reported in applications as diverse as probes for the regulation of enzyme activity and conformational studies [1,2,3], sensors and biosensors, reservoirs of metal binding motifs in metabolically stable and biologically active molecules [4,5], the targeting of peptides for molecular imaging [6,7,8], peptide drug candidates with better selectivity, activity, and lower toxicity [9,10,11,12,13], and in enzyme engineering for tuning and expanding the structural and functional features of unnatural amino acids [14,15,16] and catalysts [17] via site-specific incorporation, among many other examples.

Ion sensing is imperative in many areas, including environmental, biological, clinical, and waste management applications [18,19,20]. There is great interest in the development of artificial receptors that mimic the molecular recognition phenomenon observed in nature, which is a key event in biological processes including signaling, transport, and catalysis [18]. Specifically, interest in the development of fluorescent sensors can be explained by the distinct advantages offered by fluorescence detection in terms of sensitivity, selectivity, and fast response time. Suitable fluorescent reporters must efficiently transduce a binding event into a measurable fluorescence signal by combining a fluorophore with an analyte-responsive receptor via a saturated or unsaturated spacer [19]. The development of chemosensors for the sensing of transition metal ions is one of the most active research fields, particularly in systems that are sensitive to Cu^2+^, Pd^2+^, Hg^2+^, and Fe^3+^ [20].

Metallic cations can be complexed through N, O, and S donor atoms in natural amino acids, but synthetic manipulation at their side chain by the insertion of suitable heterocyclic systems can create novel functions as well as altered physicochemical properties, e.g., luminescence and recognition ability for metal ions and other analytes [21,22,23,24,25,26]. Considering only their photophysical properties, these unnatural amino acids can be used to assemble fluorescent, intrinsically labeled peptides without requiring additional probes [27].

Following our previous work on the synthesis and characterization of the optical properties of novel heterocyclic amino acids as well as their evaluation as efficient colorimetric/fluorescent probes for cations and anions [21,22,23,24,25,26,27], we now report the design, synthesis, and chemosensory abilities of five novel unnatural alanine derivatives bearing a benzoxazole as the reporting unit at the side chain, which is substituted at position 2 with a phenyl ring or a five-membered heterocycle (furan and thiophene) and linked to (aza)crown ether moieties of different size. Crown ethers display selectivity in complexation based on cavity and cation size and, in addition to alkaline metals, they are also effective complexing agents for alkaline-earth and transition metal ions [28]. If oxygen in the crown is replaced with softer donor atoms, such as nitrogen or sulfur, transition metals can also be complexed according to the hard and soft acids and bases (HSAB) theory [28]. The benzoxazole nucleus was chosen because of its photophysical properties (increased UV absorption and fluorescence) and also to increase the number of potential binding sites. The benzoxazole was further functionalized with electron donor heterocycles of different electronic nature (furan and thiophene) in order to modulate the response of the resulting unnatural amino acids as optical chemosensors.

Palladium is widely used in various materials, such as dental crowns, catalysts, fuel cells, and jewelry [29]. A significant quantity of palladium is released by cars to the roadside, where dust samples collected from broad-leaved plants are found to contain palladium, and rain may also wash Pd into local water systems. On the other hand, Pd-catalyzed reactions are extremely useful for the synthesis of complex molecules, although residual palladium (typically 300–2000 ppm) is often found in the product and may thus constitute a health hazard. Therefore, methods are urgently needed for the sensitive and selective detection of palladium [29].

The interaction of the novel amino acids with various alkaline, alkaline-earth, and transition metal cations was studied by means of UV-visible and fluorescence spectroscopy, through preliminary sensing studies, and spectrofluorimetric titrations in acetonitrile and aqueous mixtures.

## 2. Results and Discussion

### 2.1. Synthesis

**Figure 1:**
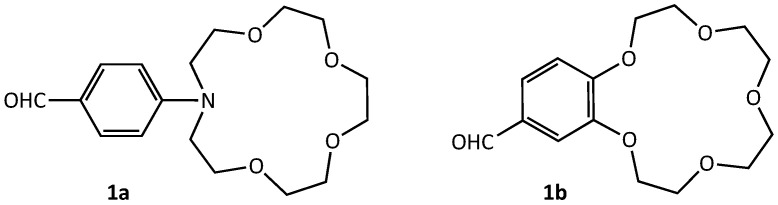
Structures of formylated azacrown ether **1a** and benzocrown ether **1b**.

**Scheme 1:**
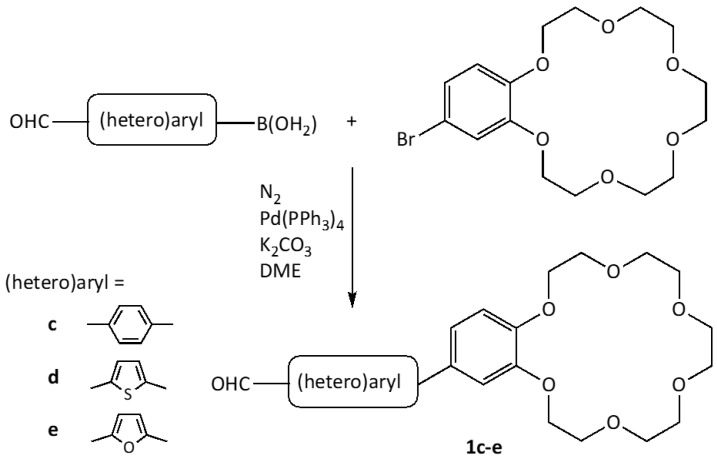
Synthesis of formyl crown ethers **1c**–**e** by Suzuki coupling.

The novel crown ether benzoxazol-5-yl-alanines **3a**–**e** were synthesized in good to excellent yields by condensation of the methyl ester of *N*-*tert*-butyloxycarbonyl-3-amino-L-tyrosine **2** and heterocyclic aldehydes **1a**–**e**, which were commercially available (**1b**, Figure 1) or prepared by Vilsmeier formylation (**1a**, Figure 1) or Suzuki coupling (**1c**–**e**, Scheme 1). The various aldehydes consisted of (aza)crown ethers of different size, functionalized with (hetero)aryls of different electronic character (phenyl, thienyl, and furyl), in order to modulate the photophysical and chemosensory properties of the resulting benzoxazol-5-yl-alanines. Furan and thiophene are electron donor heterocycles that can contribute to the overall conjugation and provide additional binding sites for cations. It was expected that additional non-covalent interactions with the O and S heteroatoms would play a synergetic role in differentiating soft transition metal cations.

Azacrown ether **1a** was obtained by Vilsmeier formylation of *N*-phenylaza-15-crown-5 with phosphorus oxychloride in *N*,*N*-dimethylformamide as a light yellow solid in 90% yield after silica gel flash chromatography, as previously published [30].

Benzo-18-crown-6 ethers **1c**–**e** were prepared by standard Suzuki coupling through the palladium-catalyzed reaction between 4′-bromobenzo-18-crown-6 ether and different (hetero) arylboronic acids, namely 4-formylphenylboronic acid, 5-formyl-2-thienylboronic acid, and 5-formyl-2-furanylboronic acid, using 1,2-dimethoxyethane (DME) as the solvent in the presence of tetrakis (triphenylphosphine) palladium(0) as the catalyst and potassium carbonate as the base (Scheme 1). Compounds **1c**–**e** were obtained in high yields after purification by silica gel column chromatography (**1c**, 91%; **1d**, 93%; **1e**, 90%).

The structures of the crown ether aldehydes **1a**,**c**–**e** were confirmed by ^1^H and ^13^C NMR spectroscopy with the characteristic formyl group signals appearing in the range of 9.61–10.00 ppm (for ^1^H) and 181.00–191.76 ppm (for ^13^C). The NMR of compound **1c** was in accordance with the previously published assignment [31].

**Scheme 2:**
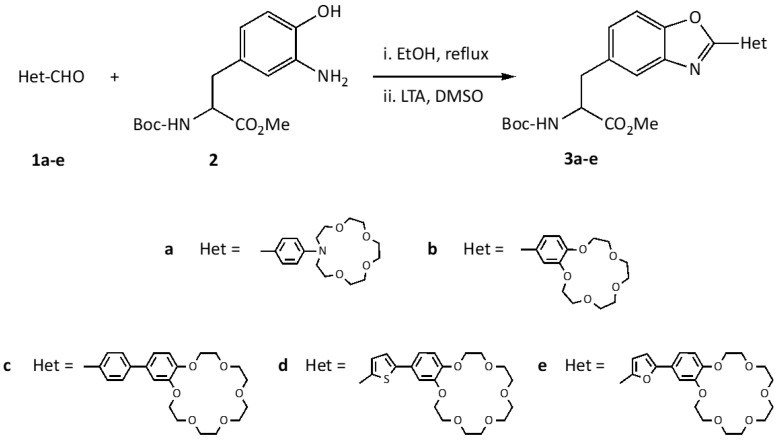
Synthesis of crown ether benzoxazolyl-alanines **3a**–**e**.

Tyrosine **2** was obtained from commercial 3-nitro-L-tyrosine by introducing adequate protecting groups at the *N*- and *C*-termini using standard procedures and reduction of the nitro group to amine, as previously published [24]. Crown ether aldehydes **1a**–**e** and tyrosine **2** were condensed in a two-step procedure: the imines were obtained by heating in ethanol under reflux and used without purification in the following oxidative intramolecular cyclization, aided by lead tetraacetate (LTA) in dimethyl sulfoxide, to afford the desired crown ether benzoxazolyl-alanines **3a**–**e** (Scheme 2).

The novel crown ether benzoxazolyl-alanines **3a**–**e** were obtained as oils in 72–96% yields and fully characterized using the usual spectroscopic techniques. The ^1^H NMR spectra of alanines **3a**–**e** showed the characteristic signals for α-H (4.60–4.67 ppm), β-CH_2_ (3.16–3.27 ppm), as well as signals due to the benzoxazole moiety. The structures were also confirmed by ^13^C NMR, with the oxazole C2 appearing between 155 and 164 ppm. In the IR spectra, the characteristic absorption bands for the NH and C=O bonds of the protecting groups (ester and urethane) were also visible, confirming that the oxidative cyclization reaction conditions did not affect the integrity of the protecting groups.

### 2.2. Photophysical Characterization

The photophysical properties of crown ether benzoxazolyl-alanines **3a**–**e** were evaluated by UV-vis absorption and fluorescence spectroscopy of degassed 1.0 × 10^−5^ M solutions in UV-grade acetonitrile [32]. The UV-Vis absorption and fluorescence data (maximum wavelength of absorption, λ_abs_; molar absorptivity, *ε*; maximum fluorescence wavelength, λ_em_; relative fluorescence quantum yield, *Φ*_F_; and Stokes’ shift, Δλ) are presented in Table 1. Relative fluorescence quantum yields were calculated using 9,10-diphenylanthracene as the standard (*Φ*_F_ = 0.95 in ethanol) [33].

Overall, benzoxazolyl-alanines **3a**–**e** showed intense fluorescence in the range of 362–445 nm with high relative quantum yields. The longer maximum wavelengths of absorption displayed by compounds **3c**–**e** were consistent with more extensive conjugation along the π-system, and the position of the absorption and fluorescence bands could have been related to the different π-bridges between the amino acid core and the crown ether unit. Thus, replacement of the phenyl group in **3c** by a thiophene in **3d** resulted in redshifted bands due to the higher electron donor character of the sulfur heterocycle [27]. A similar rationale could be made in the comparison of **3b** and **3d** (not taking into account the slight difference in the crown ring size), for which the introduction of a thiophenic π-bridge between the benzoxazole and the crown unit caused a redshift of about 50 nm in the absorption band and 80 nm in the fluorescence band. For compounds **3a**–**b**, which lacked additional π-bridges, the electron donor effect of the nitrogen at the azacrown unit was responsible for the bathochromic shift seen for **3a** compared to **3b** [30].

Comparison of the absorption and fluorescence data of the new crown ether benzoxazolyl-alanines **3a**–**e** with previously reported benzoxazolyl-alanines bearing different substituents at position 2 of the benzoxale [25,26,27] revealed that the introduction of crown ether units did not alter significantly the photophysical properties. Most importantly, the new unnatural amino acids displayed more interesting photophysical parameters due to the extended intramolecular electron delocalization and the higher push–pull character of the benzoxazole-π-bridge-crown ether system, resulting in a set of compounds that excelled in tryptophan, the most fluorescent natural amino acid (with a fluorescence quantum yield of 0.14) [34]. Therefore, this observation is of interest for the future incorporation of these unnatural amino acids into bioinspired systems such as peptide/protein structures for biological assays based on fluorescence spectroscopy, as it suggests that the interesting photophysical properties of the isolated amino acids can be preserved to yield fluorescent peptides for various applications. Our previous work in synthetic fluorescent amino acids showed that such amino acids could be incorporated into short sequences and the resulting peptides also displayed fluorescence and sensing ability [27].

**Figure 2:**
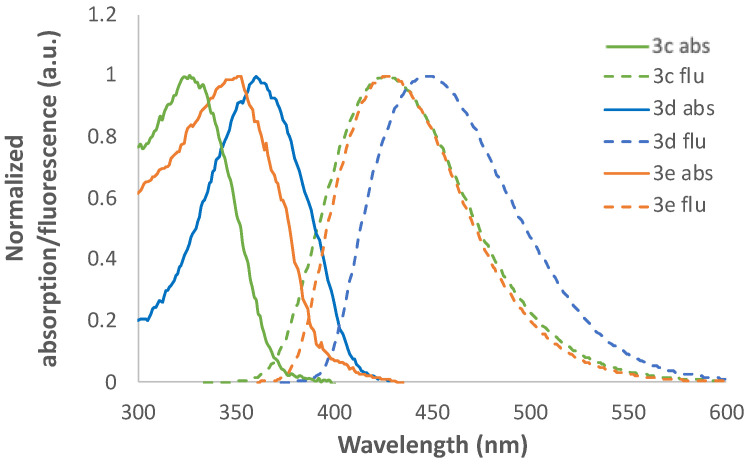
Normalized UV-visible absorption and fluorescence spectra of benzoxazolyl-alanines **3c**–**e** in 10^−5^ M solution in acetonitrile.

Figure 2 shows the normalized absorption and fluorescence spectra of benzoxazolyl-alanines **3c**–**e,** as representative examples, to better visualize the relationship between the nature of the π-bridge and the absorption and emission properties of this family of compounds.

### 2.3. Preliminary Chemosensing Studies

The novel benzoxazolyl-alanines **3a**–**e** were evaluated as fluorimetric chemosensors for the detection of different metal cations with biological and environmental relevance through preliminary chemosensory studies. These compounds consisted of a phenylalanine core modified through the introduction of an extra UV-active and highly fluorescent heterocycle at its side chain, which was expected to provide additional binding sites for a variety of ions through the heterocyclic donor oxygen, nitrogen, and sulfur atoms as well as improved photophysical properties for the chemosensing studies.

**Figure 3:**
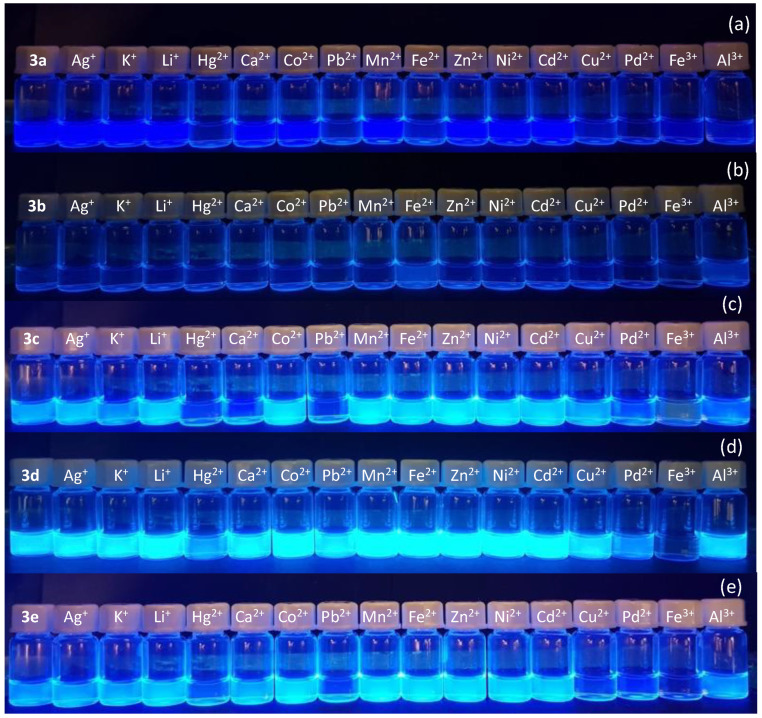
Preliminary qualitative fluorescent chemosensing study of benzoxazolyl-alanines **3a** (**a**), **3b** (**b**), **3c** (**c**), **3d** (**d**), and **3e** (**e**) in acetonitrile in the presence of 10 equivalents of each cation, visualized under a 365 nm lamp ([**3a**] = [**3c**] = [**3d**] = 3.0 × 10^−5^ M; [**3b**] = 6.0 × 10^−6^ M; [**3e**] = 1.0 × 10^−5^ M, at room temperature).

The fluorimetric behavior of compounds **3a**–**e** in the presence of selected cations was studied in acetonitrile by adding 10 equivalents of each cation to a solution of each compound. This amount is usually considered in preliminary qualitative chemosensing tests because it provides quick evidence of the sensitivity of the system being tested. Acetonitrile is an aprotic solvent widely used in these studies because it lacks the ability to establish hydrogen bonds with the analytes or sensing molecules. It was found that the compounds had different fluorimetric responses for different cations, showing a preference for interactions with mercury, lead, palladium, copper, and iron cations. Compound **3c** exhibited marked fluorescence quenching upon interaction with Hg^2+^, Pb^2+^, and Pd^2+^ and complete quenching with Fe^3+^ (Figure 3c); compound **3d** showed similar behavior, (Figure 3d); and compound **3e** showed decreased fluorescence upon interaction with Hg^2+^, Pb^2+^, and Pd^2+^ and complete quenching was seen in the presence of Cu^2+^ and Fe^3+^ (Figure 3e). Notably, compounds **3e**–**c** were able to discriminate between iron cations in different oxidation states by remarkable and selective quenching of fluorescence upon interaction with Fe^3+^ compared to interaction with Fe^2+^.

Although there was a relationship between the ionic radius of the metals and the crown cavity size (e.g., Li^+^ bound preferably to 15-C-5 while K^+^ bound to 18-C-6) [28], the additional binding sites at the various heteroatoms of compounds **3a**–**e** could be the key for the marked interaction with the transition metals.

The recognition of this type of analyte in biological and environmental media is of the utmost importance, so the development of water-soluble probes is required [35,36]. The new unnatural amino acid derivatives **3a**–**e** were poorly water soluble in their protected form and metal cations are prone to strong solvation by water, imposing an energetic barrier that inhibits sensing processes in aqueous solution. To circumvent these limitations in the fluorogenic detection of metal cations in water, instead of mixtures with pure water, a common strategy is to use surfactants [37,38]. In the case of the anionic surfactant sodium dodecyl sulfate (SDS), it has been reported that optical chemosensors as well as metal cations can be embedded into the inner hydrophobic core of SDS micelles, allowing detection of metal cations in aqueous solution by changes in fluorescence [38].

**Figure 4:**
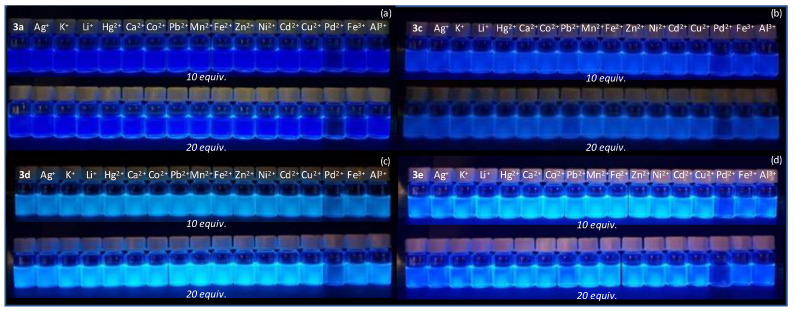
Preliminary qualitative fluorescent chemosensing study of benzoxazolyl-alanines **3a** (**a**), **3c** (**b**), **3d** (**c**), and **3e** (**d**) in SDS (20 mM, pH 7.5)/acetonitrile (90:10) in the presence of 10 and 20 equivalents of each cation, visualized under a 365 nm lamp ([**3a**] = [**3c**] = [**3d**] = [**3e**] = 1.0 × 10^−5^ M at room temperature).

Therefore, the fluorimetric response of compounds **3a**–**e** was also tested in aqueous mixtures of SDS (20 mM, pH 7.5) solution with acetonitrile, 90:10 *v*/*v*. In fact, all compounds were completely soluble at a concentration of 1.0 × 10^−5^ M. As before, changes in fluorescence were examined upon interaction with 10 and 20 equivalents of each cation. The use of a larger number of cation equivalents resulted in a more noticeable difference in the fluorimetric response. Interestingly, a selective fluorometric response was seen for Pd^2+^ in SDS aqueous solutions for compounds **3a,c**–**e** (Figure 4). Compound **3b** was not included due to low fluorescence. For compounds **3a**,**c**–**e**, the fluorescence quenching could be assigned to SDS-assisted internalization of Pd^2+^ into the inner micellar core with subsequent interaction with the fluorescent probe.

### 2.4. Spectrofluorimetric Titrations

Given the results obtained in the photophysical characterization and the preliminary chemosensing study, crown ether benzoxazolyl-alanines **3c**–**e** were chosen as representative examples and their interaction with selected cations was evaluated through spectrofluorimetric titrations in acetonitrile.

**Figure 5:**
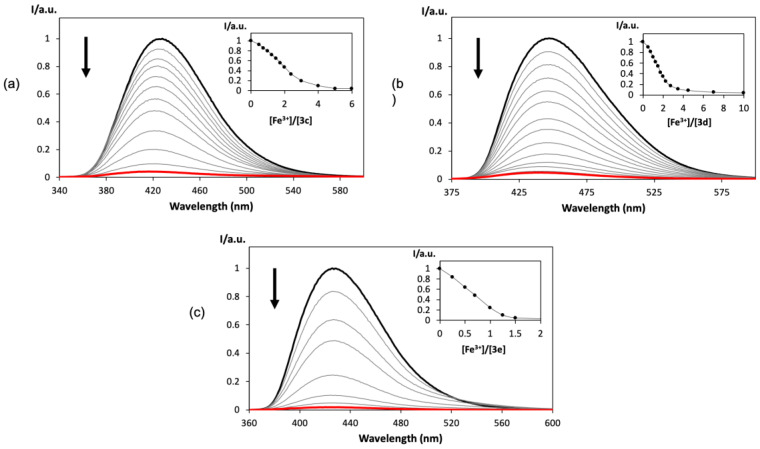
Normalized fluorimetric titrations of benzoxazolyl-alanines **3c**–**e** with Fe^3+^ in acetonitrile: (**a**) [**3c**] = 1.0 × 10^−5^ M, λ_exc_ = 324 nm. Inset: normalized emission at 428 nm as a function of added ion equivalents; (**b**) [**3d**] = 1.0 × 10^−5^ M], λ_exc_ = 363 nm. Inset: Normalized emission at 447 nm as a function of added ion equivalents; (**c**) [**3e**] = 1.0 × 10^−5^ M, λ_exc_ = 351 nm. Inset: Normalized emission at 426 nm as a function of added ion equivalents.

It was found that benzoxazolyl-alanines **3c**–**e** responded with decreased fluorescence intensity (a chelation enhancement of quenching, CHEQ effect) to increased concentrations of the tested metal cations and the sensitivity towards Fe^3+^ was evident, since the addition of a low number of metal equivalents (2–10 equiv) resulted in the complete quenching of fluorescence (Figure 5).

**Figure 6:**
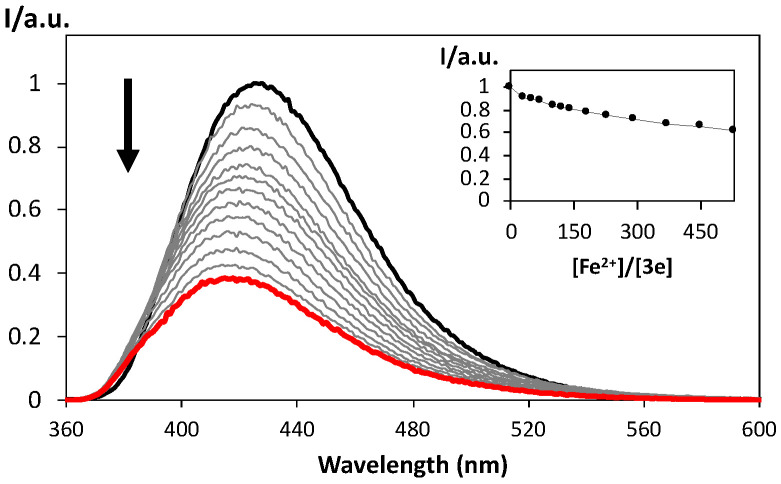
Normalized fluorimetric titrations of benzoxazolyl-alanine **3e** with Fe^2+^ in acetonitrile: [**3e**] = 1.0 × 10^−5^ M], λ_exc_ = 351 nm. Inset: Normalized emission at 426 nm as a function of added ion equivalents.

Fluorimetric titrations in acetonitrile were also conducted for compound **3e** with Fe^2+^ to confirm its ability to discriminate between Fe^3+^ and Fe^2+^. Total fluorescence quenching was not reached even after the addition of a much larger number of equivalents of the metal cation (450 equiv for *ca*. 40% quenching) (Figure 6).

**Figure 7:**
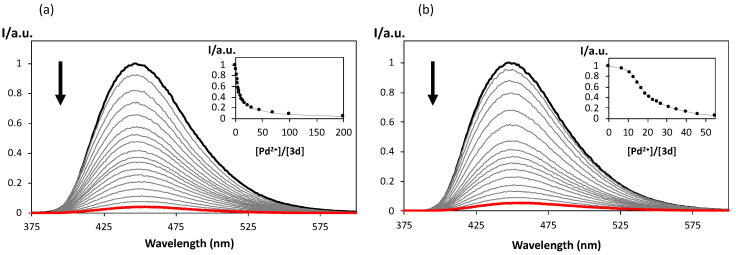
Normalized fluorimetric titrations of benzoxazolyl-alanine **3d** with Pd^2+^: (**a**) in acetonitrile and (**b**) in SDS (20 mM, pH 7.5)/acetonitrile (90:10). [**3d**] = 1.0 × 10^−5^ M], λ_exc_ = 363 nm. Inset: Normalized emission at 447 nm as a function of added ion equivalents.

Bearing in mind the behavior of benzoxazolyl-alanines **3c**–**e** in aqueous solution in the preliminary tests and the apparent selective response for Pd^2+^, the corresponding fluorimetric titrations were performed in SDS (20 mM, pH 7.5)/acetonitrile (90:10) for compound **3d**. Upon the addition of increasing amounts of Pd^2+^, complete fluorescence quenching required 50 equiv of cation (Figure 7b). In comparison with titrations with Pd^2+^ in acetonitrile, which required 200 equiv for total loss of fluorescence (Figure 7a), this can still be considered an interesting result for chemosensing in aqueous mixtures.

This chelation-induced quenching of fluorescence was in accordance with previous reports on palladium chemosensors possessing crown ether moieties, which was attributed to energy transfer quenching of the π* emissive state through low-lying, metal-centered, unfilled d-orbitals for Pd^2+^ [39]. Moreover, cooperative effects from the N and O atoms of the benzoxazole and also the O or S atoms of the furan or thiophene bridges may have been in play, also acting as binding sites for palladium.

For benzoxazolyl-alanine **3d** in SDS (20 mM, pH 7.5)/acetonitrile (90:10), the detection limit (DL) for Pd^2+^ was calculated using the equation DL = 3*σ*/slope method, where *σ* is the standard deviation of the fluorescent intensity of the analyte free solution and *S* is the slope of the linear plot of concentration-dependent fluorescence response [40]. It was found to be 5.81 μM, which was lower than the WHO threshold for palladium content in drugs [47 μM (5 ppm) to 94 μM (10 ppm)] [41].

**Figure 8:**
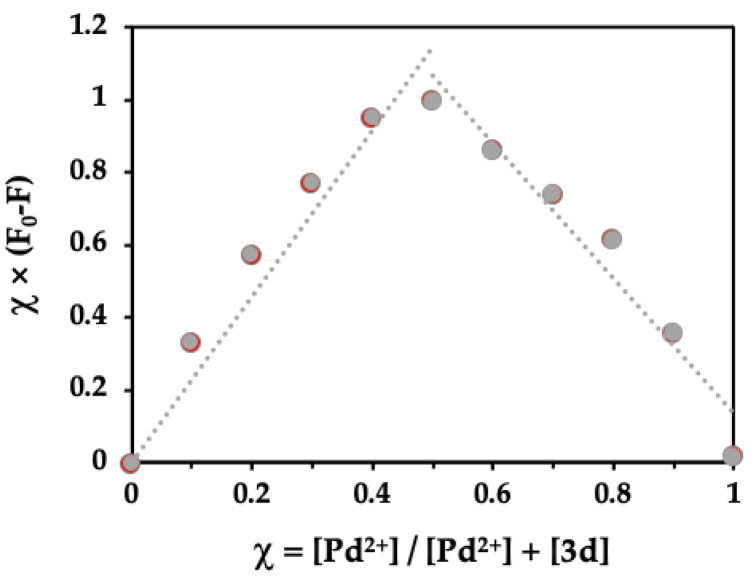
Job’s plot for the interaction of compound **3d** with Pd^2+^ by measuring the fluorescence intensity at 447 nm in SDS (20 mM, pH 7.5)/acetonitrile (90:10). The total concentration of **3d** and Pd^2+^ was 1.0 × 10^−5^ M (χ represents molar fraction, F_0_ is the initial fluorescence intensity and F is the fluorescence intensity in the presence of Pd^2+^).

In order to understand the mode of coordination between compound **3d** (as a representative example) and Pd^2+^, a Job’s plot was constructed in SDS (20 mM, pH 7.5)/acetonitrile (90:10). As can be seen in Figure 8, compound **3d** clearly formed a complex with Pd^2+^ in a 1:1 stoichiometry.

## 3. Materials and Methods

### 3.1. Synthesis General

Melting points were measured using a Stuart SMP3 melting point apparatus. TLC analyses were carried out on 0.25 mm thick precoated silica plates (Merck Fertigplatten Kieselgel 60F_254_) and spots were visualized under UV light. Chromatography on silica gel was carried out on Merck Kieselgel (230–240 mesh). NMR spectra were obtained on a Bruker Avance III 400 at an operating frequency of 400 MHz for ^1^H and 100.6 MHz for ^13^C, using the solvent peak as the internal reference at 25 °C. The solvents are indicated in parenthesis before the chemical shift values (*δ* relative to TMS and given in ppm). Assignments were supported by bidimensional heteronuclear correlation techniques. Infrared spectra were recorded on a BOMEM MB 104 spectrophotometer. Mass spectra were obtained at “C.A.C.T.I. Unidad de Espectrometria de Masas” at the University of Vigo, Spain. All commercially available reagents were used as received. We previously synthesized formylated azacrown ether **1a** and protected 3-aminotyrosine **2** [24,30].

### 3.2. Synthesis of Formyl Crown Ethers **1c**–**e** by Suzuki Coupling

General method for Suzuki coupling: In a round bottom flask under nitrogen atmosphere, 4′-bromobenzo-18-crown-6 ether (150 mg, 0.38 mmol) was dissolved in a mixture of 1,2-dimethoxyethane (6 mL) and deionized water (2 mL) at room temperature and boronic acid (0.46 mmol), tetrakis(triphenylphosphine)palladium(0) (27 mg, 0.023 mmol), and potassium carbonate (315 mg, 2.28 mmol) were added. The mixture was heated at 80 °C for about 24 h until the disappearance of the halide (checked by TLC). After cooling to room temperature, the mixture was transferred to an extraction funnel and saturated NaCl solution (10 mL) was added, followed by extraction with ethyl acetate (3 × 15 mL). The organic extracts were combined and washed with water (20 mL) and 10% aqueous NaOH (20 mL). The organic extract was dried over anhydrous magnesium sulfate, filtered, and evaporated to dryness. The crude extract was purified by column chromatography on silica gel using dichloromethane and mixtures of increasing polarity with methanol (up to DCM/MeOH, 9:1) as the eluent. The fractions containing the pure compound were combined and evaporated to dryness.

#### 3.2.1. Phenylbenzocrown **1c**

Starting from 4-formylphenylboronic acid (69 mg, 0.46 mmol), phenylbenzocrown **1c** was obtained as a light brown oil (143 mg, 91%). ^1^H NMR (400 MHz, CDCl_3_): δ 3.63*–*3.77 (m, 10H, 5 × CH_2_), 3.87*–*3.90 (m, 2H, CH_2_), 3.93*–*3.95 (m, 2H, CH_2_), 4.09*–*4.11 (m, 2H, CH_2_), 4.19*–*4.24 (m, 4H, 2 × CH_2_), 6.93 (d, J 8.0 Hz, 1H, H6*′*), 7.13 (d, J 2.0 Hz, 1H, H3*′*), 7.18 (dd, J 8.2 and 2.0 Hz, 1H, H5*′*), 7.67 (d, J 8.2 Hz, 2H, H3 and H5), 7.89 (d, J 8.2 Hz, 2H, H2 and H6), 10.00 (s, 1H, CHO) ppm. ^13^C NMR (100.6 MHz, CDCl_3_): δ 68.65 (CH_2_), 68.77 (CH_2_), 68.83 (CH_2_), 68.92 (CH_2_), 69.16 (CH_2_), 69.26 (CH_2_), 69.30 (CH_2_), 69.36 (CH_2_), 70.47 (CH_2_), 70.54 (CH_2_), 112.84 (C3*′*), 113.62 (C6*′*), 120.35 (C5*′*), 127.05 (C3 and C5), 130.14 (C2 and C6), 132.65 (C4*′*), 134.65 (C1), 146.72 (C4), 148.95 (C1*′*), 149.44 (C2*′*), 191.76 (CHO) ppm. FTIR (neat): ν 2916, 1659, 1630, 1594, 1456, 1347, 1253, 1123, 958, 867, 799 cm*^−^*^1^. MS (ESI) *m*/*z* (%): 417 (M^+^+1, 62), 416 (M^+^, 45).

#### 3.2.2. Thienylbenzocrown **1d**

Starting from 5-formyl-2-thienylboronic acid (72 mg, 0.46 mmol), thienylbenzocrown **1d** was obtained as a light yellow solid (180 mg, 93%). M.p. 175.9*–*177.1 *°*C. ^1^H NMR (400 MHz, CDCl_3_): δ 3.69*–*3.79 (m, 12H, 6 × CH_2_), 3.92*–*3.96 (m, 4H, 2 × CH_2_), 4.20*–*4.24 (m, 4H, 2 × CH_2_), 6.91 (d, J 8.4 Hz, 1H, H6*′*), 7.17 (d, J 1.6 Hz, 1H, H3*′*), 7.25 (dd, J 8.4 and 1.6 Hz, 1H, H5*′*), 7.29 (d, J 3.6 Hz, 1H, H4), 7.71 (d, J 3.6 Hz, 1H, H3), 9.86 (s, 1H, CHO) ppm. ^13^C NMR (100.6 MHz, CDCl_3_): δ 69.04 (CH_2_), 69.37 (CH_2_), 69.43 (CH_2_), 69.48 (CH_2_), 69.56 (CH_2_), 70.72 (CH_2_), 70.74 (CH_2_), 70.76 (CH_2_), 70.87 (CH_2_), 70.89 (CH_2_), 112.40 (C3*′*), 113.92 (C6*′*), 119.95 (C5*′*), 123.22 (C4), 126.36 (C2), 137.54 (C3), 141.61 (C5), 149.17 (C2*′*), 150.36 (C1*′*), 154.48 (C4*′*), 182.62 (CHO) ppm. FTIR (KBr disc): ν 2917, 1660, 1629, 1535, 1506, 1447, 1348, 1268, 1248, 956, 865, 798, 753 cm*^−^*^1^. MS (ESI) *m*/*z* (%): 423 (M^+^+1, 81), 422 (M^+^, 55).

#### 3.2.3. Furylbenzocrown **1e**

Starting from 5-formyl-2-furanylboronic acid (64 mg, 0.46 mmol), furylbenzocrown **1e** was obtained as a light brown oil (168 mg, 90%). ^1^H NMR (400 MHz, CDCl_3_): δ 3.68*–*3.73 (m, 8H, 4 × CH_2_), 3.77*–*3.79 (m, 4H, 2 × CH_2_), 3.95*–*3.98 (m, 4H, 2 × CH_2_), 4.21*–*4.23 (m, 2H, CH_2_), 4.25*–*4.28 (m, 2H, CH_2_), 6.73 (d, J 3.6 Hz, 1H, H4), 6.91 (d, J 8.4 Hz, 1H, H6*′*), 7.31 (d, J 3.6 Hz, 1H, H3), 7.34 (d, J 2.0 Hz, 1H, H3*′*), 7.39 (dd, J 8.4 and 2.0 Hz, 1H, H5*′*), 9.61 (s, 1H, CHO) ppm. ^13^C NMR (100.6 MHz, CDCl_3_): δ 68.27 (2 × CH_2_), 69.13 (2 × CH_2_), 69.54 (2 × CH_2_), 69.99 (2 × CH_2_), 70.37 (2 × CH_2_), 106.72 (C4), 110.07 (C3*′*), 113.01 (C6*′*), 119.20 (C5*′*), 122.30 (C4*′*), 124.41 (C3), 148.79 (C2*′*), 150.11 (C1*′*), 151.66 (C2), 159.64 (C5), 181.00 (CHO) ppm. FTIR (neat): ν 2923, 1665, 1594, 1488, 1455, 1357, 1253, 1212, 1122, 957, 766 cm*^−^*^1^. MS (ESI) *m*/*z* (%): 407 (M^+^+1, 92), 406 (M^+^, 68).

### 3.3. Synthesis of Crown Ether Benzoxazolyl-alanines **3a**–**e**

*General method for oxidative cyclization*: In a round bottom flask, the formylated crown ethers **1a**–**e** (1 equiv) and protected 3-aminotyrosine **2** (1 equiv) were dissolved in absolute ethanol (10 mL) and heated under reflux for 24 h. The mixture was evaporated to dryness and the imine (checked by ^1^H NMR in CDCl_3_) was obtained as a light brown oil, which did not undergo further purification. The oily imine was dissolved in DMSO (2 mL) and lead tetraacetate (3 equiv) was added, followed by stirring at room temperature for 3 days. After the addition of deionized water (10 mL), the mixture was extracted with ethyl acetate (3 × 10 mL). The organic extracts were combined, dried over anhydrous magnesium sulfate, filtered, and evaporated to dryness. The crude extract was purified by column chromatography on silica gel using dichloromethane and mixtures of increasing polarity with methanol (up to DCM/MeOH, 9:1) as the eluent. The fractions containing the pure compound were combined and evaporated to dryness (the NMR spectra are presented in the supplementary material).

#### 3.3.1. Benzoxazolyl-alanine **3a**

Starting from *N*-(4′-formylphenyl)aza-15-crown-5 **1a** (91 mg, 0.28 mmol) and protected tyrosine **2** (87 mg, 0.28 mmol), benzoxazolyl-alanine **3a** was obtained as a yellow oil (124 mg, 72%). ^1^H NMR (400 MHz, DMSO-d_6_): *δ* 1.28 (s, 9H, C(CH_3_)_3_), 2.90–3.00 (m, 2H, β-CH_2_), 3.45–3.61 (m, 20H, 10 × CH_2_), 3.61 (s, 3H, OCH_3_), 4.12–4.20 (m, 1H, α-H), 6.79 (d, *J* 8.8 Hz, 1H, NH), 7.16 (d, *J* 8.4 Hz, 1H, H6), 7.30 (d, *J* 8.4 Hz, 2H, H3′ and H5′), 7.52–7.68 (m, 3H, H4, H2′ and H6′), 7.93 (d, *J* 8.8 Hz, 1H, H7) ppm. ^13^C NMR (100.6 MHz, DMSO-d_6_): *δ* 28.14 (C(CH_3_)_3_, 36.45 (β-CH_2_), 52.13 (OCH_3_), 55.66 (α-C), 60.28 (2 × CH_2_), 67.76 (2 × CH_2_), 69.12 (2 × CH_2_), 69.62 (2 × CH_2_), 69.85 (CH_2_), 72.38 (CH_2_), 79.21 (C(CH_3_)_3_), 109.87 (C7), 111.52 (C3′ and C5′), 119.39 (C4), 120.08 (C1′), 128.89 (C6), 131.22 (C2′ and C6′), 131.67 (C5), 142.18 (C3a), 148.90 (C7a), 150.25 (C4′), 155.52 (C=O Boc), 163.64 (C2), 172.66 (C=O) ppm. FTIR (neat): *ν* 3348, 2954, 2925, 2885, 1742, 1714, 1607, 1505, 1456, 1367, 1259, 1170, 822 cm^−1^. HRMS *m*/*z* (ESI): calcd. for C_32_H_44_N_3_O_9_ 614.30721, found 614.30721.

#### 3.3.2. Benzoxazolyl-alanine **3b**

Starting from 4′-formylbenzo-15-crown-5 **1b** (107 mg, 0.37 mmol) and protected tyrosine **2** (111 mg, 0.36 mmol), benzoxazolyl-alanine **3b** was obtained as a yellow oil (180 mg, 85%). ^1^H NMR (400 MHz, CDCl_3_): *δ* 1.38 (s, 9H, C(CH_3_)_3_), 3.15–3.19 (m, 2H, β-CH_2_), 3.69 (s, 3H, OCH_3_), 3.69–3.73 (m, 8H, 4 × CH_2_), 3.88–3.90 (m, 4H, 2 × CH_2_), 4.14–4.25 (m, 4H, 2 × CH_2_), 4.58–4.61 (m, 1H, α-H), 5.08 (d, *J* 8.0 Hz, 1H, NH), 6.91 (d, *J* 8.4 Hz, 1H, H5′), 7.06 (dd, *J* 8.4 and 1.6 Hz, 1H, H6), 7.42 (d, *J* 8.0 Hz, 1H, H7), 7.46 (br s, 1H, H4), 7.69 (d, *J* 1.6 Hz, 1H, H2′), 7.77 (dd, *J* 8.4 and 1.6 Hz, 1H, H6′) ppm. ^13^C NMR (100.6 MHz, CDCl_3_): *δ* 28.14 (C(CH_3_)_3_, 38.14 (β-CH_2_), 52.16 (OCH_3_), 54.54 (α-C), 68.48 (CH_2_), 68.89 (CH_2_), 69.05 (CH_2_), 69.13 (CH_2_), 70.07 (CH_2_), 70.13 (CH_2_), 70.85 (2 × CH_2_), 79.85 (C(CH_3_)_3_), 110.15 (C7), 112.17 (C2′), 112.75 (C5′), 119.41 (C3′or C4′), 119.88 (C4), 121.51 (C6′), 125.91 (C6), 132.56 (C5), 141.94 (C3a), 148.92 (C3′ or C4′), 149.62 (C7a), 152.05 (C1′), 154.99 (C=O Boc), 163.36 (C2), 172.04 (C=O) ppm. FTIR (neat): *ν* 3333, 2952, 2869, 1750, 1688, 1563, 1537, 1478, 1436, 1352, 1278, 1135, 970, 863 cm^−1^. HRMS *m*/*z* (ESI): calcd. for C_30_H_38_N_2_NaO_10_ 609.24187, found 609.24185.

#### 3.3.3. Benzoxazolyl-alanine **3c**

Starting from phenylbenzocrown **1c** (100 mg, 0.24 mmol) and protected tyrosine **2** (74 mg, 0.24 mmol), benzoxazolyl-alanine **3c** was obtained as a light brown oil (150 mg, 89%). ^1^H NMR (400 MHz, CDCl_3_): *δ* 1.43 (s, 9H, C(CH_3_)_3_), 3.23–3.28 (m, 2H, β-CH_2_), 3.68–3.79 (m, 12H, 6 × CH_2_), 3.75 (s, 3H, OCH_3_), 3.90–3.97 (m, 4H, 2 × CH_2_), 4.13–4.28 (m, 4H, 2 × CH_2_), 4.65 (br s, 1H, α-H), 5.06 (d, *J* 8.4 Hz, 1H, NH), 6.73 (d, *J* 8.4 Hz, 1H, H6″), 7.13–7.24 (m, 3H, H6, H3″ and H5″), 7.52 (d, *J* 8.4 Hz, 1H, H7), 7.56 (br s, 1H, H4), 7.70 (d, *J* 8.4 Hz, 2H, H-3′ and H-5′), 8.29 (d, *J* 8.4 Hz, 2H, H2′ and H6′) ppm. ^13^C NMR (100.6 MHz, DMSO-d_6_): *δ* 28.14 (C(CH_3_)_3_, 42.50 (β-CH_2_), 55.60 (α-C), 57.48 (OCH_3_), 67.94 (2 × CH_2_), 68.01 (2 × CH_2_), 68.17 (2 × CH_2_), 68.51 (CH_2_), 68.55 (CH_2_), 68.65 (CH_2_), 68.71 (CH_2_), 78.44 (C(CH_3_)_3_), 111.91 (C7), 114.30 (C3″), 115.61 (C6″), 119.42 (C5″), 120.25 (C4), 123.22 (C4″), 124.67 (C6), 126.73 (C3′ and C5′), 127.13 (C2′ and C6′), 131.56 (C5), 134.61 (C1′), 141.69 (C3a), 143.29 (C4′), 147.51 (C1″), 148.42 (C7a), 149.02 (C2″), 155.54 (C=O Boc), 162.52 (C2), 172.61 (C=O) ppm. FTIR (neat): *ν* 3423, 2926, 2877, 1742, 1713, 1602, 1497, 1393, 1254, 1057, 953, 844 cm^−1^. HRMS *m*/*z* (ESI): calcd. for C_38_H_46_N_2_NaO_11_ 729.29938, found 729.29924.

#### 3.3.4. Benzoxazolyl-alanine **3d**

Starting from thienylbenzocrown **1d** (101 mg, 0.24 mmol) and protected tyrosine **2** (74 mg, 0.24 mmol), benzoxazolyl-alanine **3d** was obtained as a light brown oil (165 mg, 96%). ^1^H NMR (400 MHz, CDCl_3_): *δ* 1.42 (s, 9H, C(CH_3_)_3_), 3.20–3.23 (m, 2H, β-CH_2_), 3.69–3.77 (m, 12H, 6 × CH_2_), 3.77 (s, 3H, OCH_3_), 3.94–3.95 (m, 4H, 2 × CH_2_), 4.20–4.23 (m, 4H, 2 × CH_2_), 4.62–4.64 (m, 1H, α-H), 5.06 (d, *J* 8.0 Hz, 1H, NH), 6.89 (d, *J* 8.4 Hz, 1H, H6″), 7.09 (dd, *J* 8.0 and 1.6 Hz, 1H, H6), 7.14 (d, *J* 1.6 Hz, 1H, H3″), 7.21–7.25 (m, 2H, H5″ and H4′), 7.44 (d, *J* 8.4 Hz, 1H, H7), 7.46 (br s, 1H, H4), 7.81 (d, *J* 3.6 Hz, 1H, H3′) ppm. ^13^C NMR (100.6 MHz, DMSO-d_6_): *δ* 28.13 (C(CH_3_)_3_, 38.20 (β-CH_2_), 51.92 (OCH_3_), 55.57 (α-C), 67.53 (3 × CH_2_), 67.56 (2 × CH_2_), 68.43 (3 × CH_2_), 68.48 (2 × CH_2_), 78.41 (C(CH_3_)_3_), 109.73 (C7), 110.22 (C3″), 112.66 (C6″), 118.70 (C5″), 119.90 (C4), 124.49 (C4′), 125.52 (C6), 126.11 (C4″), 126.63 (C2′), 131.67 (C3′), 134.76 (C5), 141.60 (C3a), 148.18 (C1″), 148.62 (C2″), 148.85 (C5′), 148.89 (C7a), 155.51 (C=O Boc), 158.42 (C2), 172.58 (C=O) ppm. FTIR (neat): *ν* 3385, 2925, 1740, 1714, 1576, 1505, 1448, 1367, 1255, 1124, 956, 849 cm^−1^. HRMS *m*/*z* (ESI): calcd. for C_36_H_45_N_2_O_11_S 713.27386, found 713.27394.

#### 3.3.5. Benzoxazolyl-alanine **3e**

Starting from furylbenzocrown **1e** (114 mg, 0.28 mmol) and protected tyrosine **2** (87 mg, 0.28 mmol), benzoxazolyl-alanine **3e** was obtained as a light brown oil (173 mg, 89%). ^1^H NMR (400 MHz, CDCl_3_): *δ* 1.39 (s, 9H, C(CH_3_)_3_), 3.17–3.20 (m, 2H, β-CH_2_), 3.65–3.73 (m, 12H, 6 × CH_2_), 3.73 (s, 3H, OCH_3_), 3.81–3.92 (m, 4H, 2 × CH_2_), 4.12–4.24 (m, 4H, 2 × CH_2_), 4.58–4.60 (m, 1H, α-H), 5.09 (d, *J* 8.0 Hz, 1H, NH), 6.69 (d, *J* 3.6 Hz, 1H, H4′), 6.84–6.89 (m, 2H, H6″ and H3′), 7.09 (d, *J* 8.0 Hz, 1H, H6), 7.31 (d, *J* 2.0 Hz, 1H, H3″), 7.35 (dd, *J* 8.4 and 2.0 Hz, 1H, H5″), 7.50 (d, *J* 8.4 Hz, 1H, H7), 7.55 (d, *J* 1.2 Hz, 1H, H4) ppm. ^13^C NMR (100.6 MHz, DMSO-d_6_): *δ* 28.11 (C(CH_3_)_3_, 42.16 (β-CH_2_), 51.87 (OCH_3_), 54.87 (α-C), 67.95 (2 × CH_2_), 68.15 (CH_2_), 68.33 (2 × CH_2_), 68.66 (2 × CH_2_), 68.72 (2 × CH_2_), 68.79 (CH_2_), 78.38 (C(CH_3_)_3_), 107.40 (C4′), 109.08 (C7), 110.27 (C3″), 113.13 (C6″), 117.52 (C3′), 120.07 (C5″), 120.90 (C4), 122.03 (C4″), 126.61 (C6), 134.74 (C5), 140.32 (C2′), 141.43 (C3a), 148.17 (C1″), 148.52 (C7a), 149.07 (C2″), 154.88 (C=O Boc), 155.49 (C2), 156.81 (C5′), 172.57 (C=O) ppm. FTIR (neat): *ν* 3371, 2975, 2929, 2879, 1744, 1711, 1641, 1509, 1366, 1254, 1165, 958, 855 cm^−1^. HRMS *m*/*z* (ESI): calcd. for C_36_H_44_N_2_NaO_12_ 719.27865, obtido 719.27860.

### 3.4. Sensing Studies General

The UV-vis absorption spectra were obtained in acetonitrile solution (1.0 × 10^−5^ M) using a Shimadzu UV/2501PC spectrophotometer and the fluorescence spectra were obtained using a Horiba FluoroMax-4 spectrofluorometer.

Evaluation of benzoxazolyl-alanines **3a**–**e** as fluorimetric chemosensors was carried out in the presence of several cations (Ag^+^, K^+^, Li^+^, Hg^2+^, Ca^2+^, Co^2+^, Pb^2+^, Mn^2+^, Fe^2+^, Zn^2+^, Ni^2+^, Cd^2+^, Cu^2+^, Pd^2+^, Fe^3+^, and Al^3+^). Solutions of compounds **3a**–**e** (3.0 × 10^−5^ M) and the ions under study (1.0 × 10^−2^ M) were prepared in acetonitrile. Solutions of compounds **3a**–**e** (1.0 × 10^−5^ M) and the ions under study (1.0 × 10^−2^ M) were prepared in aqueous mixtures of SDS (20 mM, pH 7.5) solution with acetonitrile, 90:10 *v*/*v*. Preliminary studies were carried out by adding up to 10 equivalents of each cation to the solutions of compounds **3a**–**e** in acetonitrile. A similar study was performed by adding up to 10 and 20 equivalents of each cation to the solutions of compounds **3a**–**e** in an aqueous environment using SDS. The solutions were visualized in a Vilber Lourmat CN15 viewing cabinet under a UV lamp at 365 nm.

## 4. Conclusions

Considering the obtained results, it can be concluded that benzoxazolyl-alanines bearing crown ether moieties **3a**–**e** are sensitive, although not selective, fluorimetric chemosensors in acetonitrile solution, and their sensory behavior is characterized by a variable decrease in the initial fluorescence intensity upon the addition of increasing amounts of different metal cations. Alanines **3c**–**e**, which bear a benzo-18-crown-6 ether, gave a noteworthy response for trivalent iron, needing between 2 to 10 equivalents of the metal cation for complete quenching.

Bearing in mind the interest in developing chemosensors able to display selective optical responses in aqueous mixtures, solutions of alanines **3c**–**e** in aqueous SDS (20 mM, pH 7.5) with acetonitrile (90:10 *v*/*v*) demonstrated a selective response in the presence of Pd^2+^ through marked fluorescence quenching, with a low detection limit of 5.81 μM. The encouraging results in terms of photophysical and metal ion sensing properties of these compounds opens up the possibility for their use in the assembly of peptides with chemosensory/probing abilities.

## Figures and Tables

**Table 1:** UV-visible absorption and fluorescence data for benzoxazolyl-alanines **3a**–**e** in acetonitrile.

Compound	Absorption		Fluorescence			
	λ_abs_ (nm)	log *ε*	λ_em_ (nm)	*Φ*_F_	Δλ (nm) (cm^−1^)	
** 3a **	334	4.04	395	0.82	61	4624
** 3b **	310	4.03	362	0.87	52	4634
** 3c **	324	3.99	428	0.62	104	7500
** 3d **	363	4.01	445	0.69	82	5076
** 3e **	351	4.04	426	0.65	75	5016

## Data Availability

Not applicable.

## Supplementary Materials

The following supporting information can be downloaded at: https://www.mdpi.com/article/10.3390/molecules28083326/s1, ^1^H and ^13^C NMR spectra of compounds **3a**–**e**.
