# Supplementary material for: Novel Crown Ether Amino Acids as Fluorescent Reporters for Metal Ions
Source: Molecules. 2023 Apr 9;28(8):3326. doi: 10.3390/molecules28083326 (PMC10140843; doi:10.3390/molecules28083326)
Supplement: Supplementary file 1 [file molecules-28-03326-s001.zip › molecules-2223389-supplementary.pdf]

Novel Crown Ether Amino Acids as Fluorescent Reporters for Metal Ions

Patrícia M. R. Batista, Cátia D. F. Martins, M. Manuela M. Raposo * and Susana P. G. Costa *

Centre of Chemistry, Campus de Gualtar, University of Minho, 4710-057 Braga, Portugal
* Correspondence: mfox@quimica.uminho.pt (M.M.M.R.); spc@quimica.uminho.pt (S.P.G.C.)

Supplementary material

^{1}H and ^{13}C NMR spectra of compounds **3a**-**e**

Figure S1. ^{1}H NMR spectrum of compound **3a** (DMSO-d_6, 400 MHz)

Figure S2. ^{13}C NMR spectrum of compound **3a** (DMSO-d_6, 100.6 MHz)

Figure S3. ^{1}H NMR spectrum of compound **3b** ($CDCl_3$, 400 MHz)

Figure S4. ^{13}C NMR spectrum of compound **3b** ($CDCl_3$, 100.6 MHz).

Figure S5. ^{1}H NMR spectrum of compound **3c** ($CDCl_3$, 400 MHz)

Figure S6. ^{13}C NMR spectrum of compound **3c** (DMSO-d_6, 100.6 MHz)

Figure S7. ^{1}H NMR spectrum of compound **3d** ($CDCl_3$, 400 MHz)

Figure S8. ^{13}C NMR spectrum of compound **3d** (DMSO-d_6, 100.6 MHz)

Figure S9. ^{1}H NMR spectrum of compound **3e** ($CDCl_3$, 400 MHz)

Figure S10. ^{13}C NMR spectrum of compound **3e** (DMSO-d_6, 100.6 MHz)

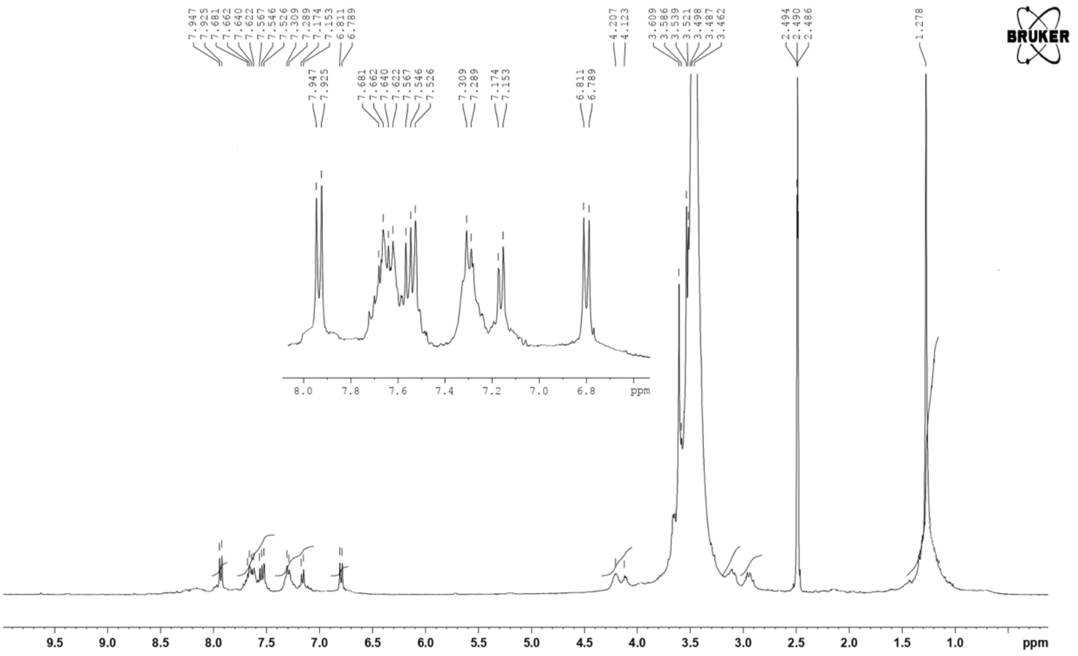

Figure S1. ^{1}H NMR spectrum of compound **3a** (DMSO-d_6, 400 MHz).

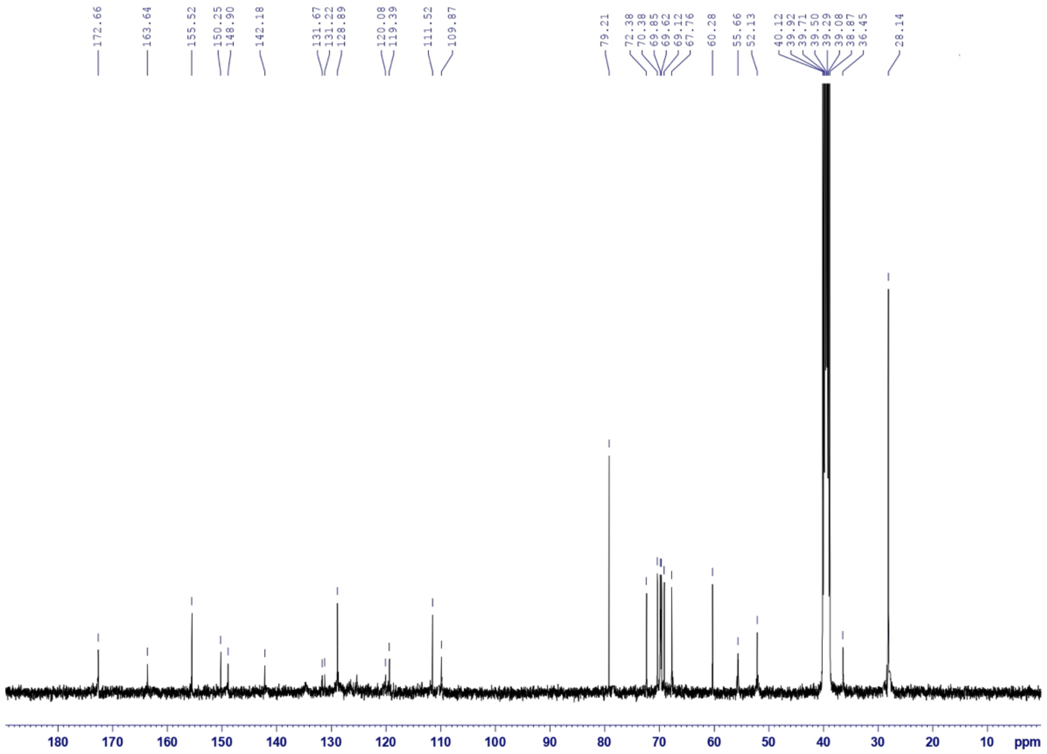

Figure S2. ^{13}C NMR spectrum of compound **3a** (DMSO-d_6, 100.6 MHz).

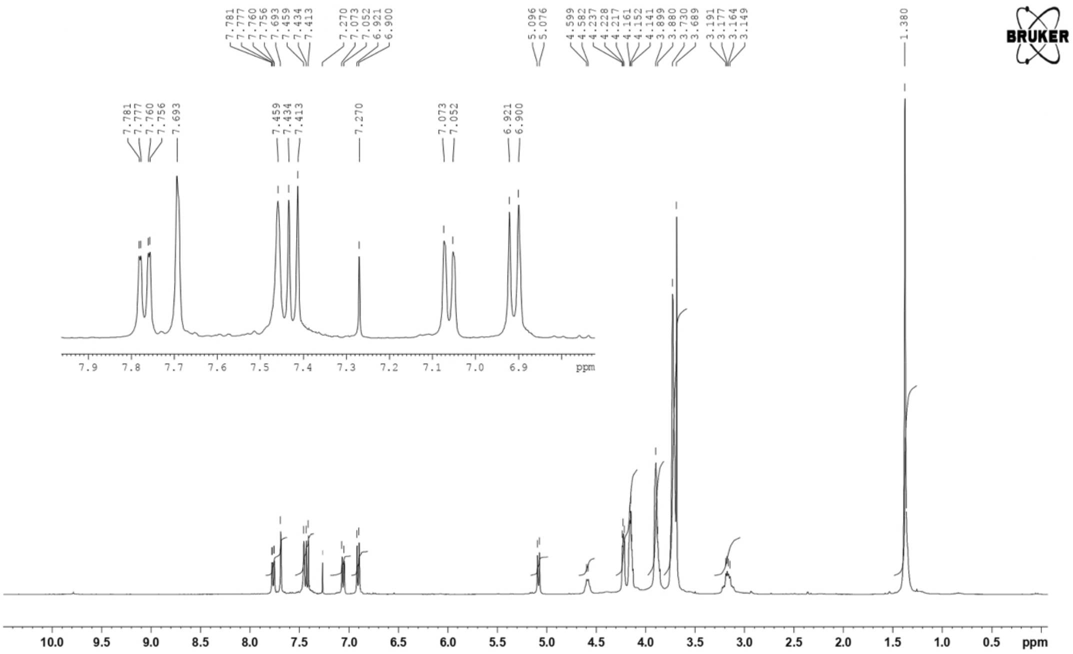

Figure S3. ^{1}H NMR spectrum of compound **3b** ($CDCl_3$, 400 MHz).

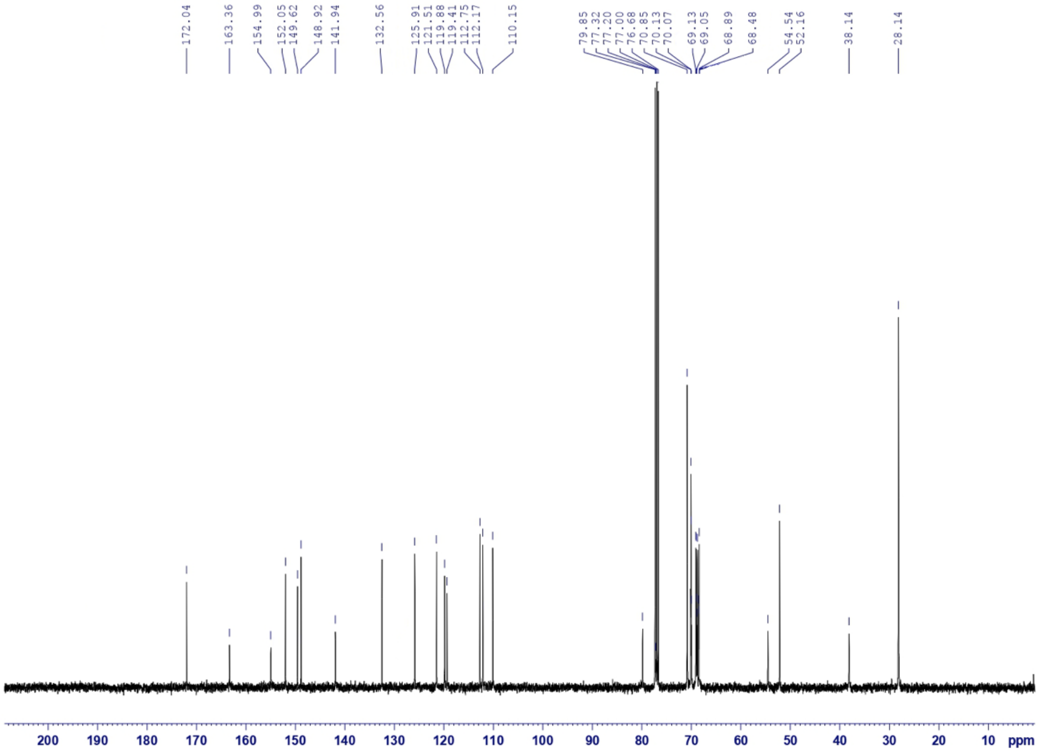

Figure S4. ^{13}C NMR spectrum of compound **3b** ($CDCl_3$, 100.6 MHz).

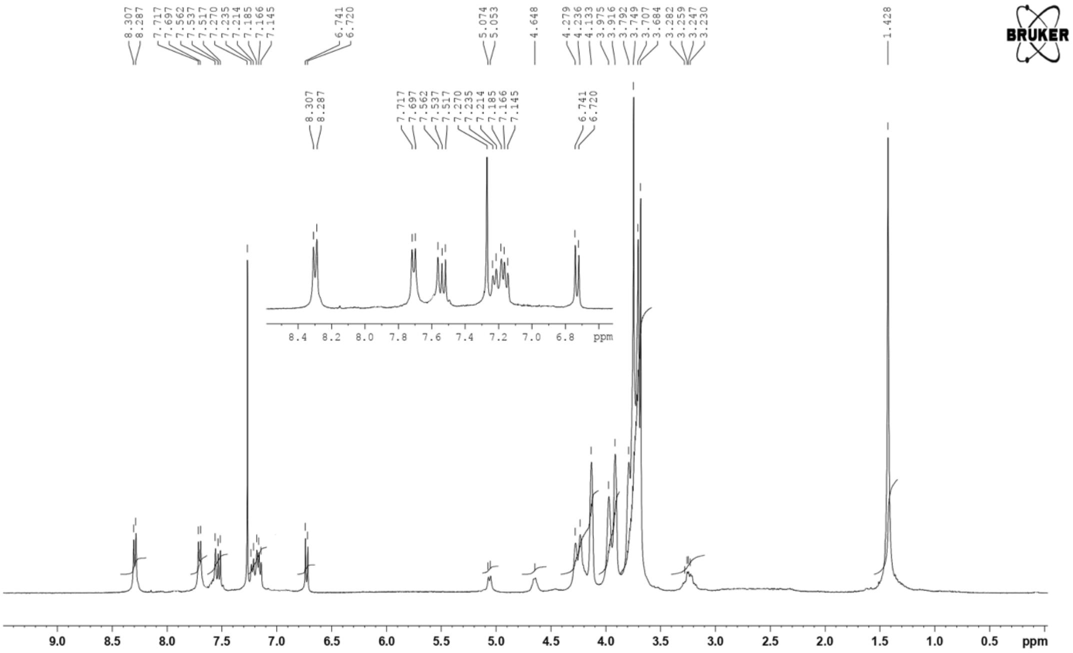

Figure S5. ^{1}H NMR spectrum of compound **3c** ($CDCl_3$, 400 MHz).

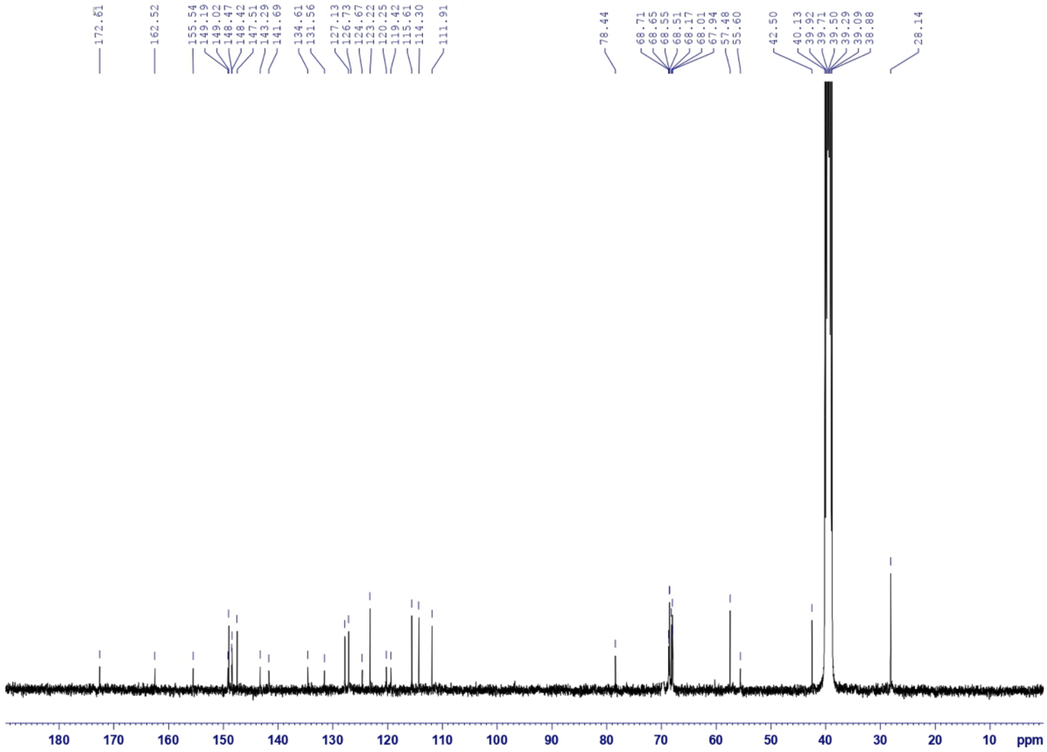

Figure S6. ^{13}C NMR spectrum of compound **3c** (DMSO-d_6, 100.6 MHz).

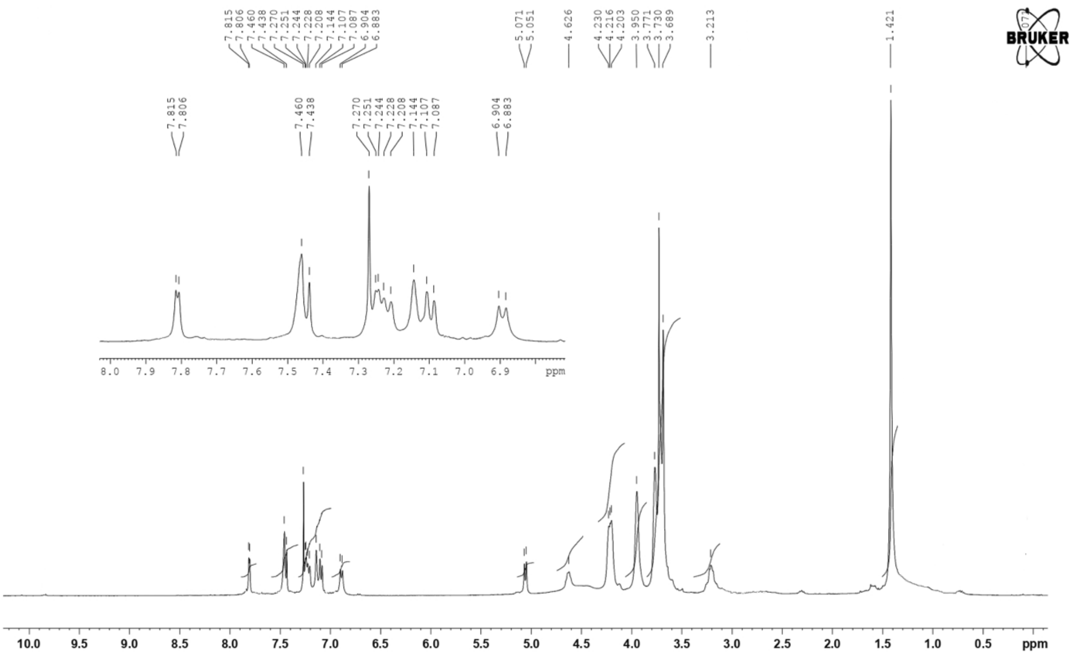

Figure S7. ^{1}H NMR spectrum of compound **3d** ($CDCl_3$, 400 MHz).

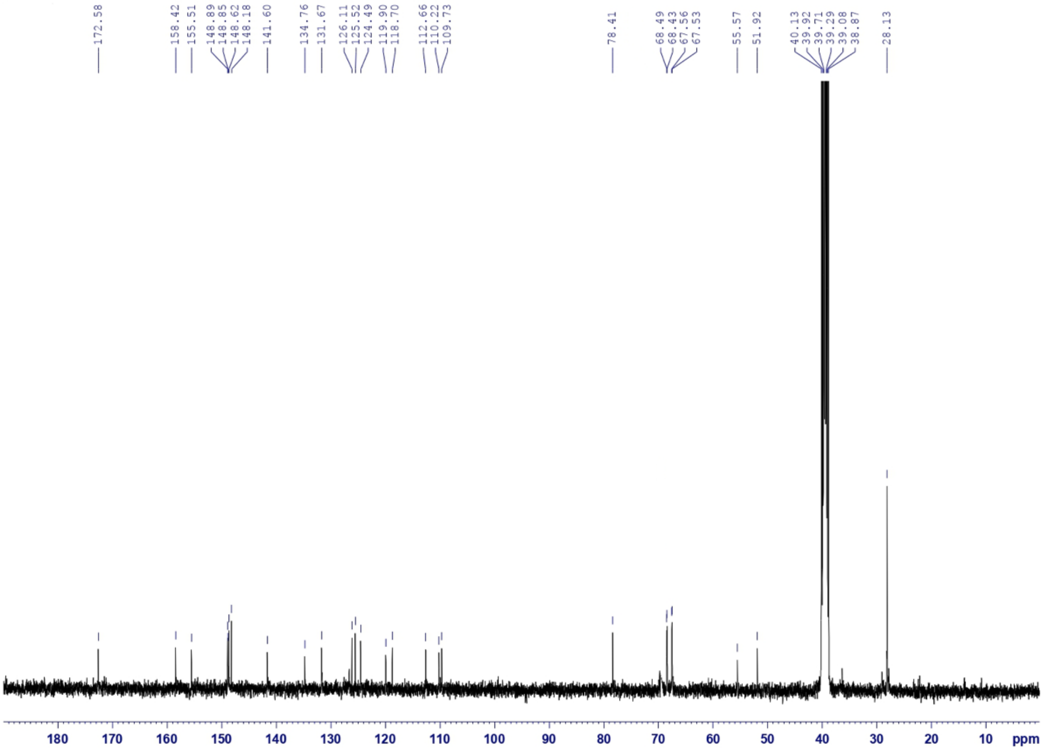

Figure S8. ^{13}C NMR spectrum of compound **3d** (DMSO-d_6, 100.6 MHz).

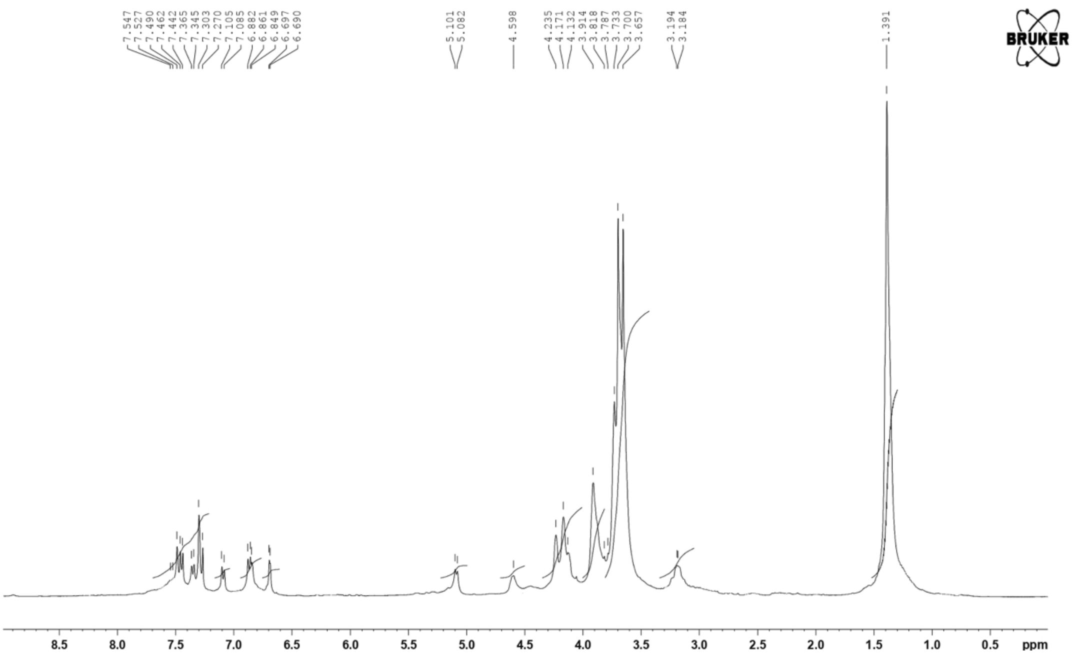

Figure S9. ^{1}H NMR spectrum of compound **3e** ($CDCl_3$, 400 MHz).

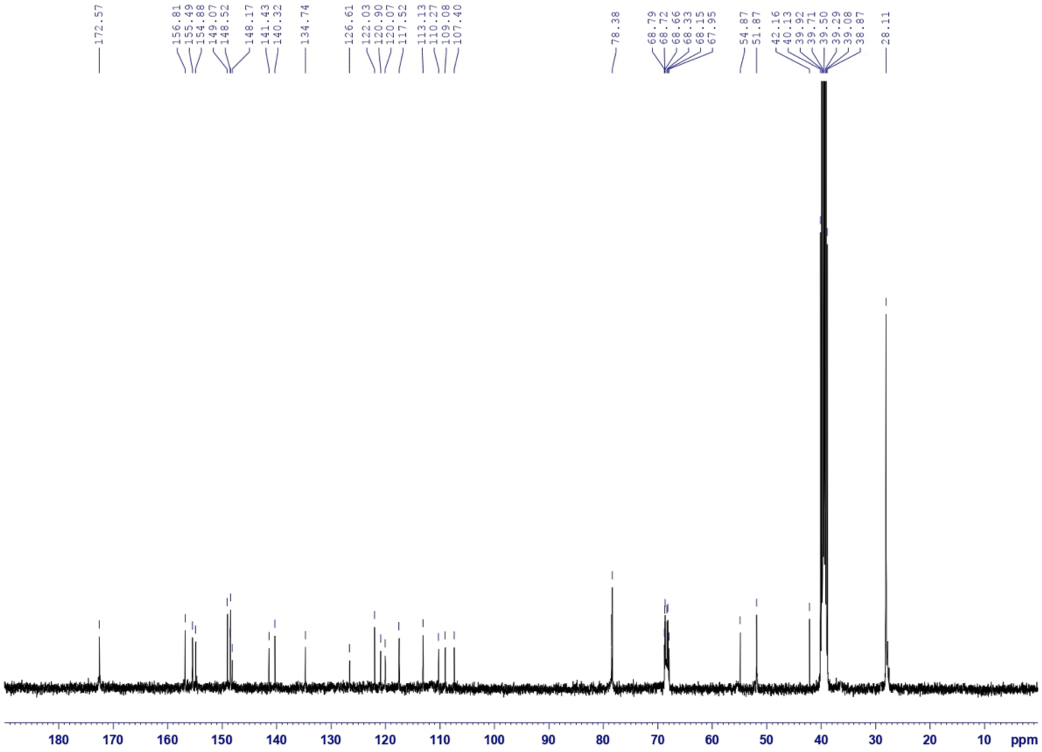

Figure S10. ^{13}C NMR spectrum of compound **3e** (DMSO-d_6, 100.6 MHz).
